# Long-term glycemic outcomes of a diabetes management platform in a traditional Chinese medicine hospital: a real-world retrospective observational study

**DOI:** 10.3389/fendo.2026.1799398

**Published:** 2026-04-23

**Authors:** Xiuming Li, Hua Wei, Wenhui Xiao, Ying Fan, Haoyue Huang, Dongyin Zou, Yuan Zhang, Yiting Wang, Lan Cheng, Tao Zou, Guanyang Zou, Guanjie Fan

**Affiliations:** 1 The Second Affiliated Hospital, Guangzhou University of Chinese Medicine, Guangzhou, China; 2 Department of Endocrinology, Guangdong Provincial Hospital of Chinese Medicine, Guangzhou, China; 3 State Key Laboratory of Dampness Syndrome of Chinese Medicine, The Second Affiliated Hospital of Guangzhou University of Chinese Medicine, Guangzhou, China; 4 State Key Laboratory of Mechanism and Quality of Chinese Medicine, Institute of ChineseMedical Sciences, University of Macau,Macao, Macao SAR, China; 5 School of Public Health and Management, Guangzhou University of Chinese Medicine, Guangzhou, China

**Keywords:** China, chronic disease management, hospital-based care, longitudinal study, real-world evidence, type 2 diabetes mellitus

## Abstract

**Background:**

Sustained glycemic control in type 2 diabetes mellitus (T2DM) remains difficult in routine care. Long-term real-world evidence on diabetes management platforms in Traditional Chinese Medicine (TCM) hospitals is limited.

**Methods:**

We conducted a real-world retrospective observational study at Guangdong Provincial Hospital of Traditional Chinese Medicine. Adults with T2DM enrolled in a hospital-based chronic disease management platform between January 2017 and December 2018 were followed for up to 36 months. Primary outcomes were longitudinal changes in glycated hemoglobin (HbA1c) and HbA1c target attainment (<7.0%). Secondary outcomes included fasting blood glucose (FBG), postprandial blood glucose (PBG), body mass index (BMI), and self-reported lifestyle and self-management behaviors. Linear mixed-effects models and generalized estimating equations were used for longitudinal analyses.

**Results:**

A total of 546 patients were included. HbA1c decreased significantly over 36 months, and the proportion achieving HbA1c <7.0% increased from 30% at baseline to 49% at 12 months and remained around 45% at 24 and 36 months. Patients with baseline HbA1c >9.0% showed the greatest absolute reductions, whereas those with baseline HbA1c <7.0% generally maintained near-target levels. Clinically meaningful HbA1c reductions were common among participants with paired measurements, and sustained HbA1c target attainment was also observed across follow-up visits. In contrast, longitudinal changes in FBG were not significant, while reductions in PBG and BMI were modest. Improvements in lifestyle and self-management behaviors were limited.

**Conclusions:**

In this TCM hospital setting, platform-based chronic disease management was associated with sustained improvement in HbA1c over 3 years. Greater benefit was observed in patients with poor baseline glycemic control, although improvements in behavioral indicators and other metabolic measures were limited.

## 1 Introduction

Type 2 diabetes mellitus (T2DM) is a prevalent, multifactorial, and currently incurable noncommunicable disease posing significant public health challenges. Diabetes affected nearly 500 million people worldwide in 2019 and is projected to reach approximately 700 million by 2045 (1). The situation is particularly severe in China, which bears one of the world’s highest diabetes burdens, with an estimated 140 million adults aged 20–79 years affected in 2021 (2). However, diabetes management in China remains suboptimal. According to a nationally representative survey (2018-2019), the awareness, treatment, and control rates among adults with diabetes were only 38.0%, 34.4%, and 33.1%, respectively (3–5). These low figures underscore that diabetes has evolved into a significant public health issue in China.

In this context, effective chronic disease management, which includes regular detection, continuous monitoring, evaluation, and comprehensive intervention for noncommunicable diseases and their risk factors, represents a cornerstone strategy for improving clinical outcomes and alleviating the diabetes burden. In practice, chronic disease management is often decentralized to the community level to enhance accessibility and reduce strain on tertiary hospitals. Studies evaluating various community-based interventions, including case management (6), team-based care (7), digital health tools (8), self-management education and support (9–12), and intensive diabetes management (13), as well as recent digital and hybrid care models (14), have demonstrated improvements in glycated HbA1c among patients with diabetes.

However, the effectiveness of chronic disease management is not limited to community settings. In China, chronic disease management has been a policy priority since the launch of the new round of Health System Reform in 2009 (15). After over a decade of exploration, various chronic disease management models have been piloted and implemented. These models range from community-based and hospital-based approaches to integrated frameworks, including general hospital-community collaborations and Centers for Disease Control-hospital-community models (16).

Despite widespread practice, chronic disease management in China continues to face systemic challenges. These include insufficient active follow-up, lack of timely evaluation of prevention and control measures, and fragmented patient health records (15, 17, 18). The effectiveness of community-based care is further limited by constraints in human resources and diagnostic capabilities, potentially reducing service quality (19, 20). Although policy encourages community-based care, tertiary hospitals in China still dominate diabetes management due to patient trust in higher-level institutions and the unequal distribution of medical resources. Consequently, many hospitals have developed their own chronic disease management (CDM) platforms to improve continuity of care (21).

This study evaluates the 36-month glycemic and behavioral outcomes of a TCM-integrated CDM platform in a major tertiary hospital in Southern China. By analyzing a cohort of 546 patients, we aimed to characterize longitudinal changes in glycemic control (HbA1c), related metabolic outcomes, and self-management behaviors in a real-world clinical setting.

## 2 Materials and methods

### 2.1 Study design and population

This real-world retrospective observational study was performed at Guangdong Provincial Hospital of Traditional Chinese Medicine. The study was designed and reported following the Strengthening the Reporting of Observational Studies in Epidemiology (STROBE) guidelines. Adult patients with confirmed T2DM who were enrolled in the hospital-based chronic disease management (CDM) platform, supported by the CDM Information System (CDMIS), between January 2017 and December 2018 were included.

Inclusion criteria were: (1) confirmed T2DM diagnosis at a specialist outpatient clinic or inpatient ward according to the Chinese Guidelines for the Prevention and Treatment of Type 2 Diabetes (2017 edition) (22), defined as fasting plasma glucose ≥7.0 mmol/L, 2-hour plasma glucose ≥11.1 mmol/L during an oral glucose tolerance test, or random plasma glucose ≥11.1 mmol/L in patients with classic symptoms of hyperglycemia; in asymptomatic patients, abnormal results were confirmed by repeat testing on a different day; (2) enrollment in the CDMIS; and (3) availability of baseline assessment data.

Exclusion criteria were: (1) cognitive impairment preventing effective communication; and (2) missing baseline HbA1c data.

A total of 546 patients met eligibility criteria and formed the full cohort (primary analysis set). Among them, 112 patients with available HbA1c measurements at all key follow-up visits (baseline and months 3, 6, 9, 12, 24, and 36) comprised the completer cohort for sensitivity analyses. Ethical approval was granted by the Ethics Review Committee of Guangdong Provincial Hospital of Traditional Chinese Medicine (Approval No. B2016-091-01; June 2016). All patients provided written informed consent upon enrollment, including permission for research use of de-identified clinical data.

### 2.2 CDM platform of Guangdong provincial hospital of traditional Chinese medicine

The CDM platform at Guangdong Provincial Hospital of Traditional Chinese Medicine was established in 2009. It is a hospital-based, multidisciplinary program coordinated across multiple clinical departments. An interdisciplinary team comprising specialist physicians, nurses, nutrition professionals, psychologists, and rehabilitation staff operates the platform. It integrates inpatient specialty wards and outpatient chronic disease clinics. Within specialty wards, designated physicians and nurses with relevant expertise provide care to enrolled patients. Operational management is supported by a dedicated team consisting of senior and attending physicians, full-time nurses, and data-entry clerks responsible for routine documentation and data management within the CDMIS.

Care delivery follows a tiered approach. Patients are stratified by condition severity and complexity, and clinical management and follow-up plans are assigned accordingly. Follow-up was primarily conducted through in-person clinic visits, with routine documentation and scheduling supported by the platform.

In 2017, the platform launched an updated CDMIS that integrates hospital medical resources and supports centralized chronic disease data infrastructure. Core CDMIS modules include patient information management, scheduling and reminders, health education, individualized care plan documentation, standardized assessment management and data capture, and statistical reporting and analysis. The system supports routine clinical workflows such as data entry and retrieval, clinician documentation of individualized care plans, task-based reminders for healthcare staff, follow-up task management, and data export for analysis. Currently, the platform covers more than 30 chronic conditions, and the diabetes management team was among the first teams established.

### 2.3 Diabetes management measures

The diabetes management team implemented standardized treatment and chronic care procedures incorporating both conventional diabetes care and TCM elements. The main components are detailed below.

#### 2.3.1 Medication management

Patients were prescribed pharmacotherapy according to the Chinese Guidelines for the Prevention and Treatment of Type 2 Diabetes (2017 edition) (22). Medication counseling included dosing schedules, administration methods, insulin injection techniques (site selection and precautions), and adherence support. Chinese patent medicines or individualized herbal decoctions were prescribed based on TCM syndrome differentiation when clinically indicated.

#### 2.3.2 Diet

Diabetes specialists and nutrition professionals jointly formulated individualized dietary plans guided by the dietary therapy principles in the Chinese Guidelines for the Prevention and Treatment of Type 2 Diabetes (2017 edition) and the *TCM Constitution Classification and Determination Standard* (2009). Plans emphasized balanced energy intake and adequate nutrition, tailored to individual clinical status, dietary habits, regional characteristics, seasonality, and constitutional type. Patients received counseling on meal planning and practical TCM-oriented dietary therapy recipes. A “Diabetic Diet Education Manual” was developed to facilitate patient understanding and implementation.

#### 2.3.3 Exercise coaching

Exercise counseling followed the exercise therapy principles of the Chinese Guidelines for the Prevention and Treatment of Type 2 Diabetes (2017 edition) and the *2013 China Diabetes Exercise Treatment Guidelines*, considering TCM constitution recommendations (23). Patients were encouraged to engage in at least 30 minutes daily of moderate-intensity activity (e.g., jogging, brisk walking, cycling, or swimming), with individualized intensity adjustments as appropriate. Traditional exercise modalities for health preservation, such as the 24-form Simplified Tai Chi and Ba Duan Jin, were also recommended.

#### 2.3.4 Health education

Health education was delivered through multiple formats, including printed educational materials, group lectures, and patient exchange meetings. A small-group counseling session was held every two weeks, covering diet, exercise, and diabetes-related knowledge. Monthly lectures addressed key diabetes management topics, including seasonal TCM healthcare practices and precautions. These activities aimed to enhance disease awareness and promote adherence to treatment and self-management.

#### 2.3.5 Blood glucose monitoring guidance

Patients were advised and supported to record FBG and PBG at least monthly as routine self-monitoring, with frequency adjustments based on clinical status. HbA1c testing was typically conducted in the hospital approximately every 3 months, although intervals could be extended (e.g., to 6–12 months) for clinically stable patients, consistent with routine practice.

#### 2.3.6 *TCM* therapy

TCM characteristic therapies were offered based on patient needs and clinical conditions. TCM dietary guidance was provided according to TCM constitution assessment. For patients with abdominal adiposity, traditional abdominal massage and acupoint massage were used as supportive therapies. For patients experiencing numbness or pain in the feet, TCM foot massage techniques were applied as adjunctive care to relieve symptoms.

#### 2.3.7 Regular follow-up

Follow-up schedules were individualized based on patient condition, typically categorized as 3-month, 6-month, or annual plans. Clinicians documented individualized care plans in the CDMIS. The system generated automatic reminders for healthcare staff to complete scheduled follow-up tasks.

### 2.4 Primary and secondary outcomes

#### 2.4.1 Primary outcome

The primary outcomes were glycated HbA1c levels and achievement of HbA1c targets at key follow-up visits (baseline and months 3, 6, 9, 12, 24, and 36). Adequate glycemic control was defined as HbA1c <7.0%, consistent with clinical guidelines. HbA1c was measured at baseline and approximately every three months thereafter as part of routine hospital testing. Testing intervals were adjusted according to patients’ glycemic status and clinic visits. HbA1c results at months 12, 24, and 36 were emphasized for reporting purposes.

#### 2.4.2 Secondary outcomes

Secondary outcomes included FBG, PBG, BMI, and lifestyle/self-management behaviors. FBG, PBG, and body weight were primarily collected from patient home-monitoring records (finger-stick glucose and weight measurements) and documented at each clinic visit using the “Diagnosis Evaluation Form for Medical Visits”.

### 2.5 Data collection and entry

At baseline, patients completed the “Diabetes Chronic Disease Management Form, “capturing demographic and clinical information (age, diabetes duration, education level, diagnosis, diabetes-related complications, hypertension, smoking, alcohol use, prior treatment, and payment method such as medical insurance coverage). At each clinic visit, patients completed the “Evaluation Form for Medical Visits, “recording self-management and health status indicators (glycemic control, blood pressure, weight, glucose monitoring, diet, exercise, psychological status, and medication adherence).

Data entry and management were performed by dedicated staff. Data-entry clerks entered patient-completed forms using the CDMIS scale/assessment entry functions and imported laboratory results from hospital systems. Full-time nurses provided health education, used CDMIS reminder functions to ensure planned follow-ups, guided patients in completing self-administered forms, and reviewed content completeness. Physicians provided outpatient care, clinical assessments, and documented individualized management plans. The clinical team regularly reviewed and exported CDMIS data for completeness and quality assurance.

### 2.6 Statistical analysis

Analyses were performed using SPSS version 27.0. Baseline characteristics were summarized as mean (standard deviation, SD) for continuous variables and number (percentage) for categorical variables. Group comparisons, when applicable, used independent-samples t-tests for continuous variables and chi-square tests or Fisher’s exact tests for categorical variables.

Linear mixed-effects models were fitted separately for HbA1c, FBG, PBG, and BMI to evaluate longitudinal changes. Time was modeled as a categorical fixed effect (baseline as reference), and a random intercept for participant ID was included to account for within-subject correlation. Models were estimated using restricted maximum likelihood.

GEE with a binomial distribution and logit link were used to analyze binary outcomes (HbA1c target attainment [<7.0%] and predefined binary lifestyle and health behaviors). Time was treated as a categorical variable (baseline as reference) with an exchangeable working correlation structure and robust standard errors. Analyses used all available observations under the modeling assumptions of linear mixed-effects models and GEEs. All tests were two-sided, and statistical significance was set at α = 0.05.

Sensitivity analyses were conducted in the completer cohort (n=112) with HbA1c data available at all key follow-up visits.

## 3 Results

### 3.1 Enrollment and baseline characteristics

Between January 2017 and December 2018, 546 patients with T2DM were enrolled in the CDM platform at Guangdong Provincial Hospital of Traditional Chinese Medicine.

At baseline, the mean age was 59.0 years, and the mean diabetes duration was 9.11 years. Approximately half of participants were male, and most had previously received antidiabetic treatment. Diabetes-related complications and hypertension were common. Detailed baseline demographic and clinical characteristics of the cohort are shown in **Table 1**.

**Table 1:** Baseline characteristics comparing the entire cohort (n=546) and the completer cohort (n=112).

Characteristic	N=546	N=112	*P* value
Sex (n, %)			0.602
Male	268 (49)	58 (52)	
Female	278 (51)	54 (48)	
Age (mean ± SD)	59.0 ± 14.2	57.5 ± 15.3	0.318
Illness duration (mean ± SD)	9.11 ± 6.22	9.85 ± 5.79	0.433
Waist to hip ratio (mean ± SD)	0.93 ± 0.07	0.92 ± 0.06	0.245
Education (n, %)			0.073
College and above	158 (29)	41 (37)	
Junior and senior school	290 (53)	60 (53)	
Primary school and below	96 (18)	11 (10)	
Complication (n, %)			0.405
None	125 (23)	22 (20)	
<3	170 (31)	42 (37)	
>=3	251 (46)	48 (43)	
Hypertension (n, %)			0.825
No	278 (51)	58 (52)	
Yes	266 (49)	53 (48)	
Received treatment previously (n, %)			0.626
No, newly diagnosed	97 (18)	18 (16)	
Yes, received medication	449 (82)	93 (84)	
Payment method (n, %)			0.046
Medical insurance	418 (77)	94 (84)	
Free medical service	66 (12)	15 (13)	
Self-financed medical	55 (10)	3 (3.0)	
Other	7 (1.0)	0 (0.0)	

To characterize follow-up completeness, the number of participants with HbA1c measurements ateach follow-up visit (0, 3, 6, 9, 12, 24, and 36 months) was summarized (**Supplementary Table 1**). Baseline characteristics were compared between participants with at least one follow-up HbA1c measurement and those without follow-up data to assess potential selection bias (**Supplementary Table 2**). Because the group without follow-up HbA1c data was small (n=6), selection bias cannot be ruled out.

A subgroup of 112 participants had HbA1c measurements at all key follow-up visits (0, 3, 6, 9, 12, 24, and 36 months). Baseline characteristics were compared between this “completer subgroup” and the full cohort to descriptively assess follow-up completeness. Characteristics were broadly similar between groups, except for payment method (medical insurance, free medical service, self-financed medical, or other), which differed significantly (P<0.05).

### 3.2 Glycemic outcomes over follow-up

In the full cohort (n=546), linear mixed-effects model analyses revealed a significant association between follow-up time and HbA1c levels (P<0.001). Mean HbA1c decreased from 9.15% (95% CI: 8.92-9.38) at baseline to 7.21% (95% CI: 6.98-7.27) at 12 months, 7.26% (95% CI: 7.04-7.39) at 24 months, and 7.00% (95% CI: 6.82-7.24) at 36 months (**Table 2**).

**Table 2:** Glycemic control indicators at baseline and at 12-, 24-, and 36-month follow-ups (n = 546 patients).

Indicator	Time point	Mean ± SD	Adjusted mean (95% CI)	Change from baseline (95% CI)	*P* value
HbA1c (%)	Baseline	9.15 ± 2.74	9.15 (8.92, 9.38)	Ref.	
	Month 12	7.21 ± 1.34	7.13 (6.98, 7.27)	-2.02 (-2.26, -1.78)	<0.001
	Month 24	7.26 ± 1.72	7.22 (7.04, 7.39)	-1.93 (-2.20, -1.67)	<0.001
	Month 36	7.00 ± 2.00	7.03 (6.82, 7.24)	-2.12 (-2.42, -1.82)	<0.001
FBG (mmol/L)	Baseline	7.24 ± 1.88	7.32 (7.05, 7.58)	Ref.	
	Month 12	7.23 ± 1.51	7.28 (7.08, 7.48)	-0.04 (-0.31, 0.23)	0.772
	Month 24	7.30 ± 1.90	7.23 (6.99, 7.28)	-0.08 (-0.44, 0.27)	0.644
	Month 36	7.30 ± 2.02	7.29 (7.07, 7.52)	-0.02 (-0.33, 0.28)	0.878
PBG (mmol/L)	Baseline	9.41 ± 2.49	9.42 (9.05, 9.78)	Ref.	
	Month 12	9.31 ± 2.22	9.32 (9.00, 9.64)	-0.10 (-0.56, 0.36)	0.674
	Month 24	8.67 ± 3.26	8.52 (8.05, 9.00)	-0.89 (-1.48, -0.30)	0.003
	Month 36	8.57 ± 3.21	8.56 (8.18, 8.93)	-0.86 (-1.34, -0.38)	<0.001
BMI (kg/m^2^)	Baseline	24.1 ± 4.47	24.1 (23.7, 24.5)	Ref.	
	Month 12	24.1 ± 4.23	24.1 (23.5, 24.7)	0.05 (-0.55, 0.66)	0.859
	Month 24	22.9 ± 5.32	23.1 (22.3, 23.8)	-1.03 (-1.85, -0.21)	0.014
	Month 36	23.1 ± 5.16	23.1 (22.5, 23.7)	-1.01 (-1.67, -0.34)	0.003

HbA1c: (F (3, 354.393) = 91.970, p < 0.001; FBG:(F (3, 183.025) = 0.086, p =0.968; PBG:(F (3, 215.084) = 5.649, p < 0.001; BMI:(F (3, 232.739) = 4.185, p = 0.007.

Consistent with these findings, GEE showed a significant time effect on HbA1c target attainment (HbA1c <7.0%, P<0.001). The proportion achieving glycemic targets increased from 30% (95% CI: 26-34%) at baseline to 49% (95% CI: 44-54%) at 12 months, remaining around 45% at 24 and 36 months (**Table 3**). In primary analyses, time effects for FBG were not significant, whereas modest but significant reductions in PBG and BMI were observed at later time points (**Table 2**).

**Table 3:** HbA1c control rates at baseline and at 12-, 24-, and 36-month follow-ups (n = 546 patients).

Time point	Control rate (95% CI)	Change from baseline (95%CI)	OR (95% CI)	P value
Baseline	0.30 (0.26, 0.34)	Ref.	Ref.	
Month 12	0.49 (0.44, 0.54)	0.20 (0.12, 0.27)	2.325 (1.841, 2.935)	<0.001
Month 24	0.45 (0.40, 0.51)	0.16 (0.08, 0.24)	1.979 (1.532, 2.556)	<0.001
Month 36	0.45 (0.39, 0.50)	0.15 (0.07, 0.24)	1.931 (1.481, 2.517)	<0.001

HbA1c control rate: Wald χ^2^ = 54.157, df = 3, p < 0.001.

**Figure 1:**
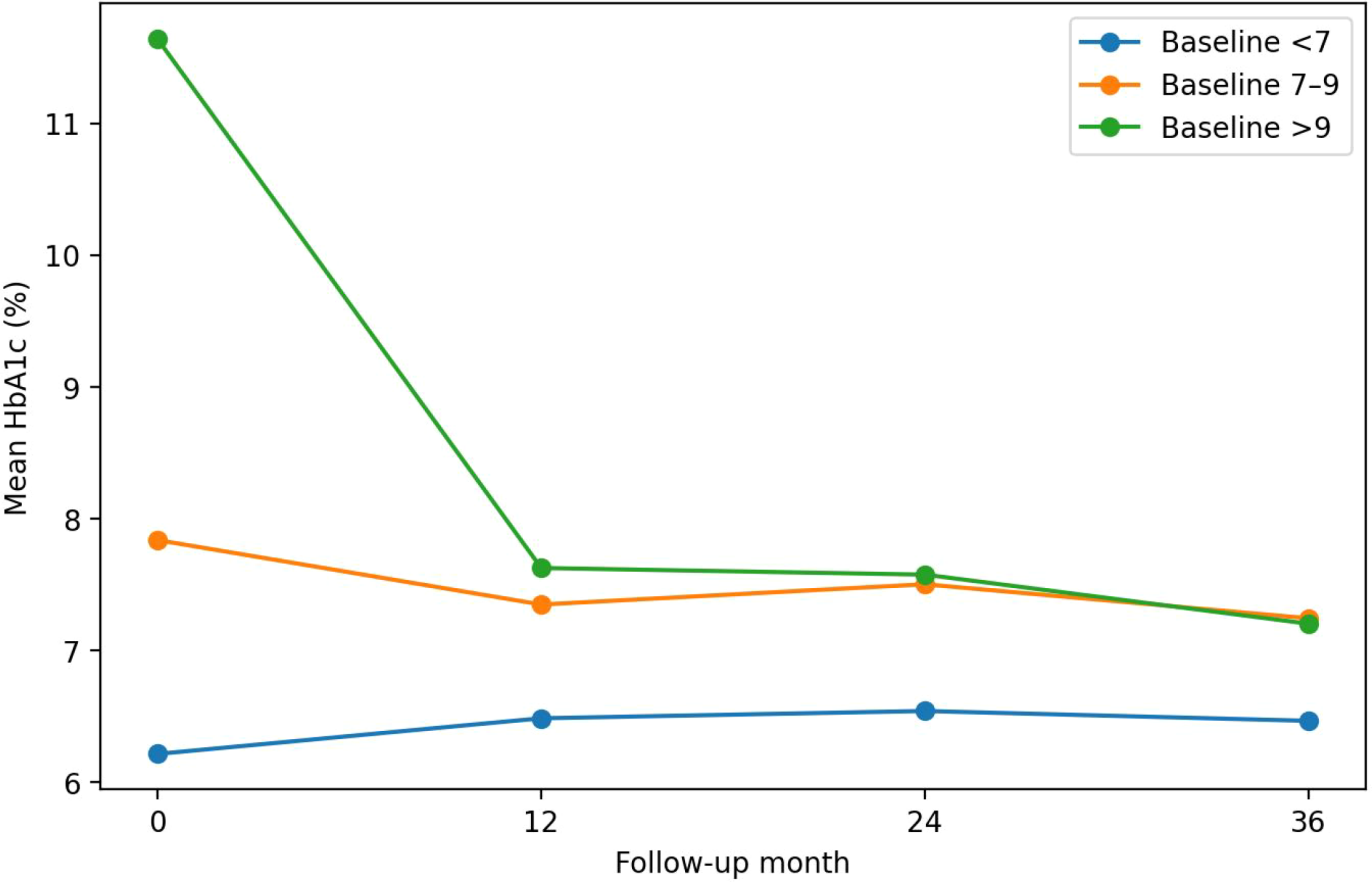
Mean HbA1c trajectories during follow-up stratified by baseline HbA1c (<7.0%, 7.0–9.0%, >9.0%).

In stratified analyses by baseline HbA1c (<7.0%, 7.0-9.0%, >9.0%), participants with baseline HbA1c >9.0% experienced the largest absolute reductions during follow-up, while those with baseline HbA1c <7.0% generally maintained levels near target (**Figure 1**; **Table 4**). Improvements in HbA1c target attainment were primarily observed among participants with baseline HbA1c ≥7.0% (**Table 4**). To assess clinical relevance, the proportions achieving HbA1c reductions ≥1.0% (and ≥2.0%) from baseline at 12, 24, and 36 months among participants with paired measurements are shown in **Table 5A**. Sustained glycemic control (HbA1c <7.0%) across multiple follow-up visits is summarizedin **Table 5B**.

**Table 4:** HbA1c trajectories and control rates during follow-up stratified by baseline HbA1c.

Baseline HbA1c stratum	HbA1c at 0 mo, mean ± SD (n)	Control rate <7% at 0 mo	HbA1c at 12 mo, mean ± SD (n)	Control rate <7% at 12 mo	HbA1c at 24 mo, mean ± SD (n)	Control rate <7% at 24 mo	HbA1c at 36 mo, mean ± SD (n)	Control rate <7% at 36 mo
<7	6.22 ± 0.48 (n=140)	100.0% (n=140)	6.49 ± 0.73 (n=96)	77.1% (n=96)	6.54 ± 1.48 (n=92)	65.2% (n=92)	6.47 ± 1.41 (n=84)	65.5% (n=84)
7–9	7.84 ± 0.61 (n=154)	0.0% (n=154)	7.35 ± 1.21 (n=94)	39.4% (n=94)	7.50 ± 1.37 (n=84)	39.3% (n=84)	7.25 ± 2.16 (n=85)	40.0% (n=85)
>9	11.64 ± 1.98 (n=246)	0.0% (n=246)	7.63 ± 1.56 (n=140)	35.7% (n=140)	7.58 ± 1.91 (n=138)	39.9% (n=138)	7.20 ± 2.19 (n=125)	42.4% (n=125)

**Table 5A:** Clinically meaningful HbA1c improvements and sustained glycemic control during follow-up. HbA1c reductions from baseline among participants with paired measurements.

Month	N (paired HbA1c)	HbA1c reduction ≥1.0% (%)	HbA1c reduction ≥2.0% (%)	Mean change (baseline–follow-up, %)
12.0	330.0	46.7	36.4	1.76
24.0	314.0	51.0	39.8	1.83
36.0	294.0	51.4	39.8	2.03

**Table 5B:** Sustained glycemic control (HbA1c <7.0%) across follow-up visits.

Definition	N (required HbA1c)	Sustained control (%)
Sustained control at 12 & 24 months	235	33.2
Sustained control at 12 & 36 months	205	31.2
Sustained control at 12, 24 & 36 months	161	24.8

### 3.3 Lifestyle and health behaviors during follow-up

In the full cohort (n=546), GEE analyses showed significant changes over time for dietary controland self-monitoring of blood glucose (SMBG) (both P<0.001). No significant longitudinal changes were observed for exercise frequency, mental status, or medication adherence (**Table 6**). GEE models evaluated outcomes using predefined binary contrasts (diet: “good dietary control” vs. others; exercise: “every day” vs. others; SMBG: >1 time/week vs. others; mental state: “good” vs. others; medication adherence: “regular” vs. others).

The proportion reporting good dietary control increased from 81% at baseline to 89% at 12 monthsbut declined to 70% at 24 months and 65% at 36 months (Wald χ^2^ = 33.773, P<0.001). Similarly, the proportion performing SMBG more than once per week decreased from 80% at baseline to 74% at 12 months, and further to 60% at 24 and 36 months (Wald χ^2^ = 51.082, P<0.001). In contrast, time effects were not statistically significant for exercising every day (Wald χ^2^ = 6.586, P = 0.086), reporting a good mental state (Wald χ^2^ = 2.540, P = 0.468), or regular medication adherence (Wald χ^2^ = 3.060, P = 0.382) (**Table 6**).

**Table 6:** Lifestyle and health behaviors at baseline and at 12-, 24-, and 36-month follow-ups (n = 546 patients).

Indicators	Baseline	Month 12	Month 24	Month 36	Wald χ²	P value
Diet (n, %)					33.773	<0.001^a^
Good dietary control	217 (81)	229 (89)	123 (70)	189 (65)		
Poor dietary control	9 (3.0)	11 (4.0)	6 (3.4)	21 (7.2)		
Occasional overeating	42 (16)	47 (16)	49 (28)	81 (28)		
Exercise (n, %)					6.586	0.086^b^
Everyday	223 (48)	140 (48)	84 (47)	120 (41)		
>5 times /week	108 (24)	67 (23)	36 (20)	71 (24)		
<4 times /week	89 (19)	65 (22)	42 (24)	74 (25)		
Never	40 (9.0)	20 (7.0)	17 (9.5)	30 (10)		
Self-test blood glucose (n, %)						
>1 time /week	368 (80)	222 (74)	107 (60)	176 (60)	51.082	<0.001^c^
>1 time /month	72 (16)	70 (23)	65 (36)	107 (36)		
<1 time /month	20 (4.0)	7 (2.0)	7 (3.9)	12 (4.1)		
Mental state (n, %)						
Good	319 (69)	211 (71)	123 (69)	197 (67)	2.540	0.468^d^
Slightly anxious	81 (18)	53 (18)	35 (20)	56 (19)		
Anxious	11 (2.0)	6 (2.0)	5 (2.8)	12 (4.1)		
Insomnia	49 (11)	28 (9.0)	16 (8.9)	30 (10.2)		
Medication (n, %)					3.060	0.382^e^
Regular	378 (83)	232 (79)	137 (77)	240 (82)		
Sometimes missed	70 (15)	58 (20)	36 (20)	48 (16)		
Often missed	7 (2.0)	3 (1.0)	4 (2.2)	4 (1.4)		

Generalized estimating equation (GEE) models evaluated predefined binary contrasts: a, Diet: good dietary control vs. others; b, Exercise: everyday vs. others; c, Self-test blood glucose frequency: >1 time/week vs. others; d, Mental state: good vs. others; e, Medication: regular vs. others.

### 3.4 Sensitivity analyses

**Figure 2:**
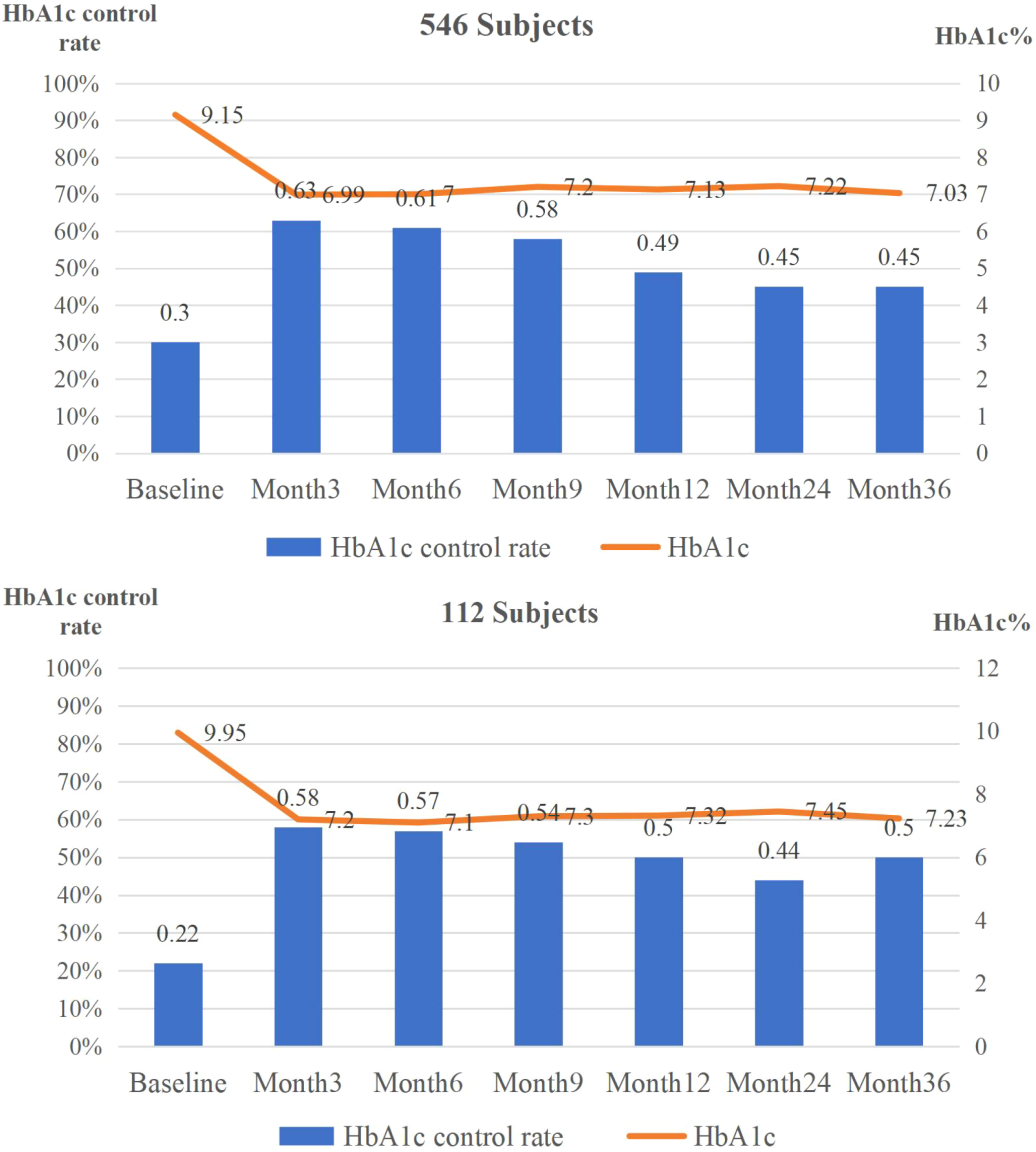
Primary outcomes at baseline and at months 3, 6, 9, 12, 24, and 36 (full and completer cohorts).

**Figure 3:**
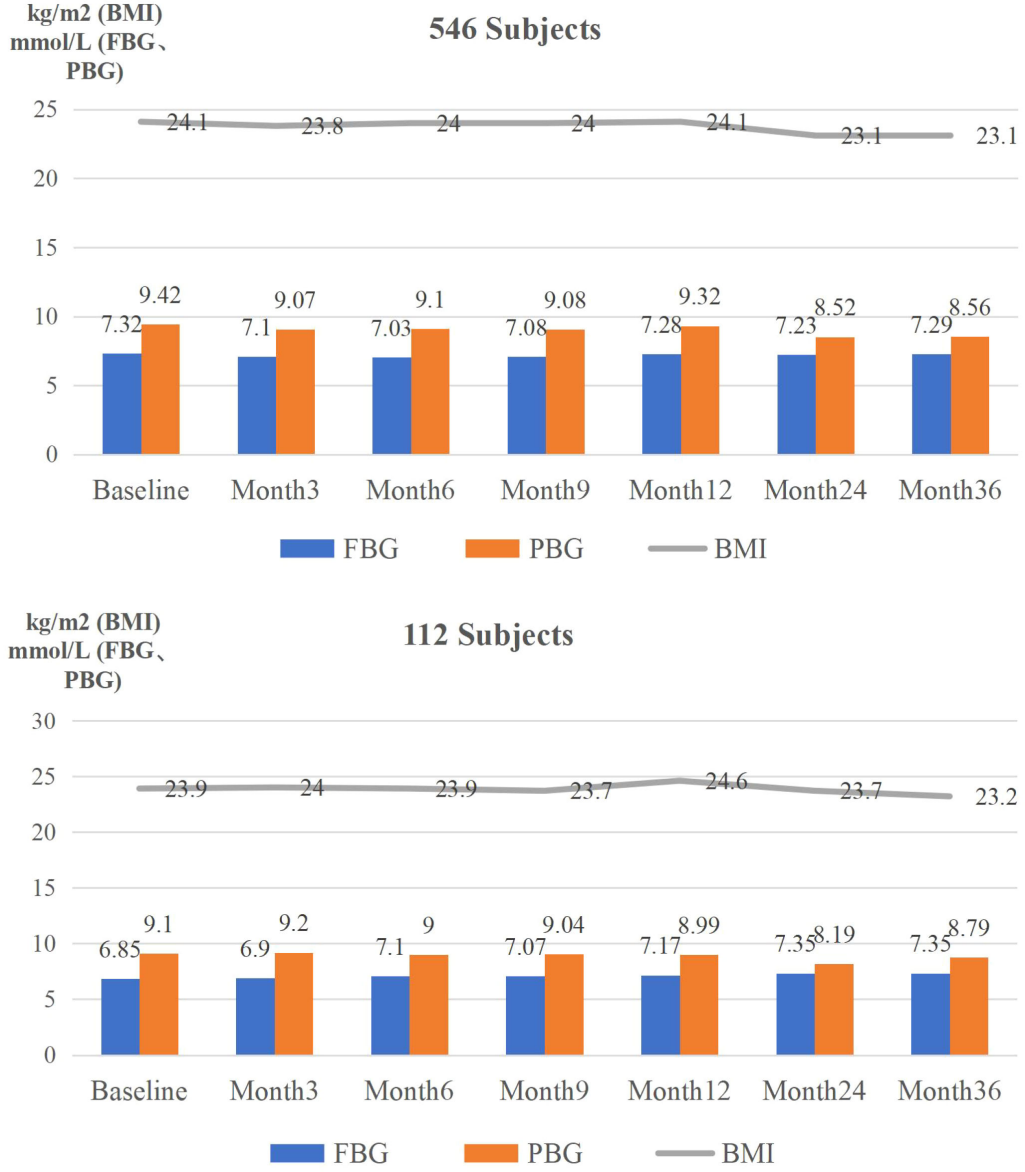
Secondary outcomes at baseline and at months 3, 6, 9, 12, 24, and 36 (full and completer cohorts).

**Figure 4:**
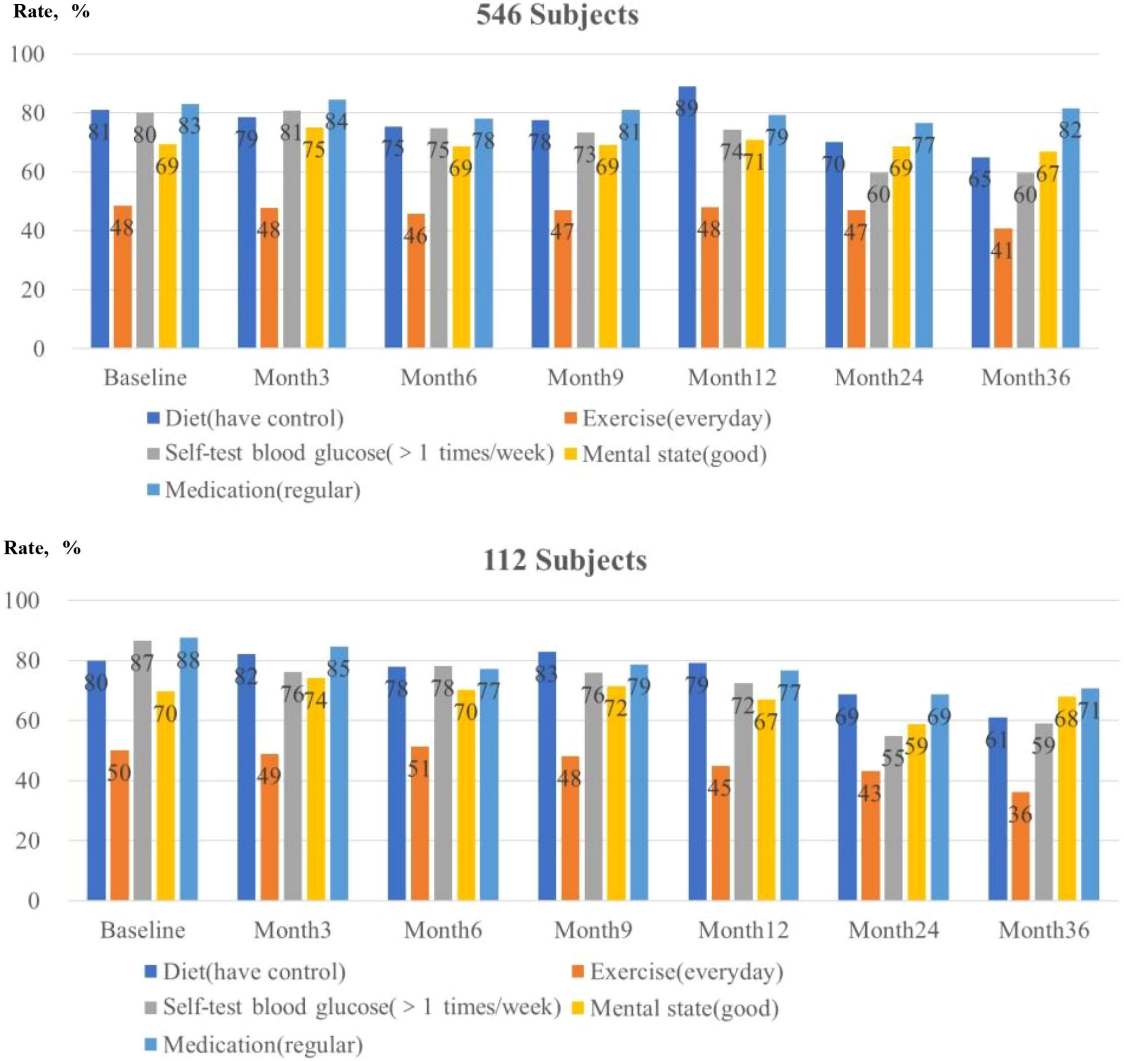
Lifestyle and health behaviors at baseline and at months 3, 6, 9, 12, 24, and 36 (full and completer cohorts).

Sensitivity analyses were conducted in a subgroup of 112 participants with HbA1c measurements available at all follow-up visits (0, 3, 6, 9, 12, 24, and 36 months). At baseline, the completer subgroup had higher mean HbA1c than the full cohort (9.95% vs. 9.15%, P = 0.006), while the proportion achieving HbA1c <7.0% did not differ significantly (22.3% vs. 29.1%, P = 0.127). Within this subgroup, HbA1c levels decreased during follow-up, and HbA1c target attainment increased, consistent with the primary analysis (**Supplementary Tables 3**, **4**). Overall, the direction and pattern of glycemic outcomes and health-related behaviors were similar to the full cohort (**Supplementary Table 5**; **Figures 2**–**4**).

**Figure 5:**
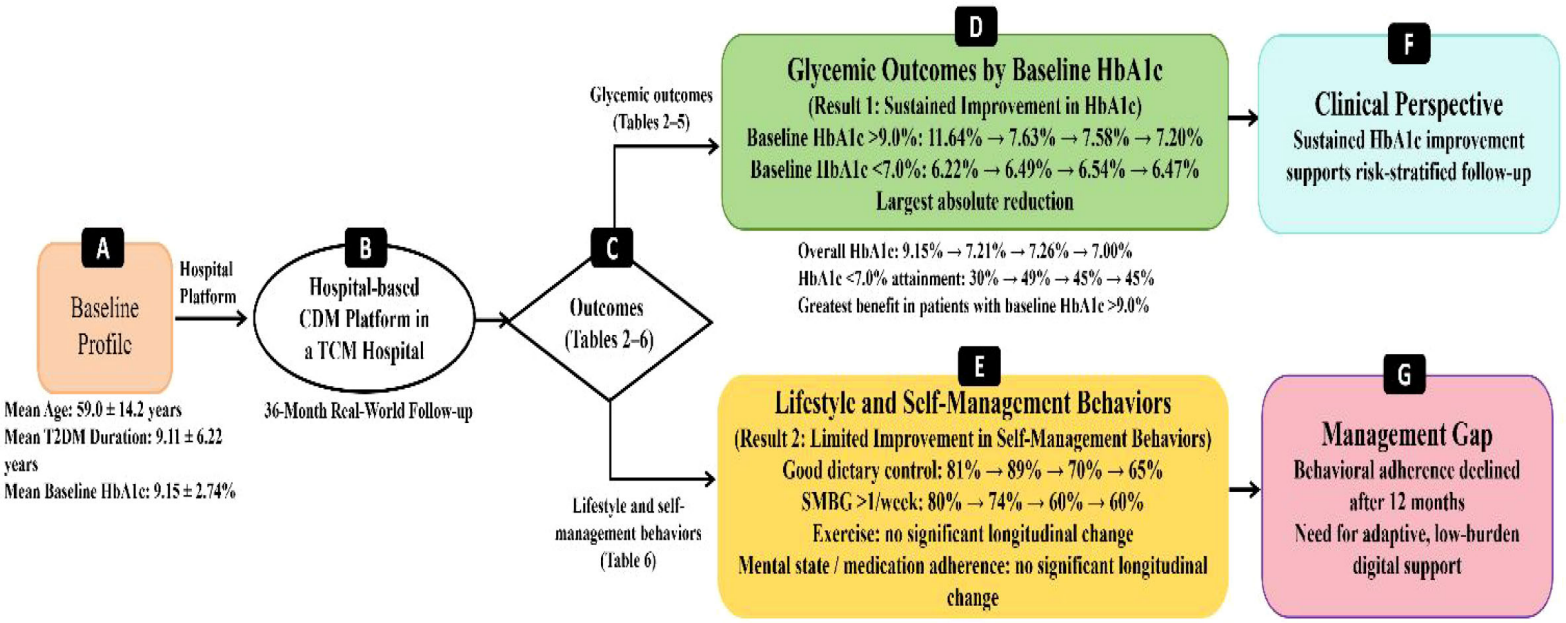
Summary of the main findings of the study.

To provide an integrated overview of the main findings from **Tables 1**-**6**, an additional summary figure was developed to illustrate the baseline profile, longitudinal glycemic outcomes, lifestyle and self-management behaviors, and their clinical implications (**Figure 5**).

## 4 Discussion

### 4.1 Summary of findings

In this retrospective, real-world observational study, glycemic outcomes and behavioral factors were assessed among patients with type 2 diabetes managed through a hospital-based CDM platform in a TCM hospital in Guangdong, China. By including 36-month follow-up data, this study adds valuable evidence on long-term glycemic trajectories within this under-studied context, where real-world evaluations of diabetes management platforms in TCM hospitals remain limited.

A notable improvement in glycemic control was observed during follow-up. Specifically, mean HbA1c decreased over 36 months, and a higher proportion of patients achieved HbA1c <7.0% compared with baseline. The greatest absolute reductions in HbA1c occurred in patients with poor baseline control (HbA1c >9.0%). Clinically meaningful HbA1c improvements, such as reductions ≥1.0% and sustained target attainment across visits, were common among patients with paired measurements. In contrast, lifestyle behaviors and other metabolic indicators showed only modest improvements. For some behaviors, adherence declined after 12 months, suggesting challenges in maintaining long-term self-management.

Although the observed HbA1c reduction appears larger than that reported in some diabetes management studies, these results should be interpreted cautiously given the observational design and the lack of a concurrent control group. Potential explanations include higher baseline HbA1c levels, allowing greater room for improvement, intensified clinical attention early in follow-up, and regression to the mean. Moreover, selective follow-up, with outcome measurements more frequently available among certain patients, as well as differences in case-mix or care processes, may also contribute, rather than the effects of any single intervention component.

In contrast to HbA1c, fasting and postprandial glucose did not show consistent longitudinal improvements. This discrepancy may partly be due to differences in measurement context and frequency. HbA1c was measured in the hospital at approximately 3-month intervals, reflecting average glycemic exposure over the preceding 2–3 months and being less influenced by short-term fluctuations. As a well-established marker of long-term glycemic control, HbA1c remains central to diabetes assessment, although it does not fully capture short-term glycemic variability or postprandial excursions (24). By contrast, fasting and postprandial glucose values in the present study were self-reported by patients on a monthly basis. Such measurements may be more susceptible to daily variability in diet, physical activity, emotional state, timing of tests, and differences in measurement conditions among individuals. Consequently, continuous glucose monitoring might provide a more comprehensive understanding of daily glycemic patterns than sporadic self-monitoring measurements (25, 26).

Despite structured education and regular follow-up, lifestyle behaviors showed limited improvement over time, with certain behaviors declining after the first year. In particular, fewer patients reported maintaining good dietary control and performing SMBG more than once weekly after 12 months. This indicates ongoing difficulties in sustaining self-management behaviors during long-term care. Such behavioral trends might contextualize the plateau observed in glycemic target attainment after 12 months, although causal inferences cannot be established from the current study design. These findings underscore the need for ongoing reinforcement and more adaptable follow-up strategies, potentially supported by digital health tools (e.g., tailored reminders, feedback loops, and frequent, low-burden interactions).

### 4.2 Strengths and limitations of the platform and study

This study has several strengths. First, it represents routine clinical practice in a tertiary TCM hospital and provides longitudinal real-world data on diabetes management over a 36-month follow-up period. Second, the multidisciplinary care model implemented by the platform involved physicians, nurses, and nutrition specialists, aligning with contemporary CDM practices. Third, the integrated clinical information system allowed continuous tracking of key outcomes and follow-up indicators, enabling comprehensive evaluation of glycemic trajectories and related health behaviors.

Several limitations must be acknowledged. From a platform perspective, care was predominantly hospital-centered, with limited integration of community-based services. This approach may have reduced continuity of care for patients with stable conditions and constrained the platform’s scalability. Follow-up relied largely on face-to-face encounters, potentially reducing flexibility, contributing to decreased adherence in some self-management behaviors, and leading to incomplete outcomes data over time. Additionally, the platform did not include mobile health functionalities or real-time glucose data integration (e.g., continuous glucose monitoring). This absence might have restricted the capacity to provide continuous, low-burden support and timely feedback between clinical visits.

From a research standpoint, this observational study lacked a concurrent control group, and exposure to specific platform components was neither standardized nor quantified. Consequently, the observed improvements cannot be definitively attributed to the platform or any single component. Furthermore, the individual contributions of specific elements, including TCM-related practices, could not be separated from co-interventions and standard clinical care. Residual confounding, regression to the mean, and selective follow-up remain possible despite descriptive assessments of follow-up completeness. These limitations must be considered when interpreting the magnitude and persistence of observed glycemic improvements.

### 4.3 Comparison with literature

Previous evaluations of diabetes management programs generally reported modest HbA1c improvements, typically ranging between approximately 0.5% and 1.0%, often with follow-up periods shorter than one year (6, 7, 9–12). In this real-world cohort, we observed larger HbA1c reductions and relatively stable HbA1c maintenance over a longer duration. Differences in baseline risk profiles and care intensity may partly explain this contrast. Higher baseline HbA1c levels provided greater potential for improvement and were commonly associated with larger absolute reductions, while early-phase education and medication adjustments likely contributed to initial improvements. Nevertheless, due to the observational design and absence of a control group, cross-study comparisons require cautious interpretation and do not imply causal platform effects.

Our findings extend existing literature by highlighting heterogeneity in glycemic trajectories according to baseline HbA1c. Participants with poor baseline control (HbA1c >9.0%) experienced the most significant HbA1c reductions, whereas those with baseline HbA1c <7.0% generally maintained near-target levels during follow-up. From a service-delivery perspective, this pattern supports adopting a pragmatic, risk-stratified approach. Patients with poorer baseline control might benefit from intensive early support and medication optimization, whereas those near-target could receive low-burden maintenance and relapse-prevention strategies. For patients with poor initial control, hospital-based follow-up might be especially beneficial for treatment intensification and multidisciplinary care. In contrast, once stable control is achieved, follow-up could shift to primary care or community clinics, where easier access and lower visit burden may encourage sustained self-management. Our findings clarify the strategic positioning of tertiary hospitals within hierarchical medical systems. Although CDM is increasingly decentralized to primary care, tertiary hospitals remain essential for high-risk or difficult-to-manage populations. Thus, tertiary hospitals’ primary role should evolve from routine follow-up to high-intensity intervention for complex cases and serving as “educators and trainers” for community care providers. By empowering primary care providers through specialized training and standardized management protocols, tertiary institutions can reserve specialized resources for patients with the greatest clinical needs and simultaneously improve overall diabetes care across the healthcare system. Future studies should examine whether referral pathways enhance continuity and outcomes. Such a step-up/step-down pathway could enhance efficiency by aligning care intensity with clinical need. Beyond mean changes, our study reported clinically meaningful endpoints, including proportions achieving HbA1c reductions of ≥1.0% (and ≥2.0%) and sustained glycemic control across multiple visits. These indicators might offer more actionable benchmarks for program evaluation and patient counseling than average HbA1c differences alone, guiding tailored follow-up intensity.

Consistent with prior literature, HbA1c improvements did not accompany sustained enhancements in lifestyle behaviors. Dietary control and SMBG tended to decline after 12 months, despite overall glycemic improvement. This pattern suggests a potential two-phase trajectory in routine platform-based care: an initial phase of intensified clinical attention and improved glycemia, followed by a maintenance phase in which behavioral adherence decreases. This apparent “decoupling” between stable HbA1c and declining self-management behaviors might partially be explained by the “metabolic memory” established during early intensive management, where initial glycemic optimization confers long-term legacy benefits. Importantly, reduced SMBG frequency may be appropriate once glycemic targets are achieved or treatment regimens stabilize, aligning with guideline-oriented practices (27). However, declining dietary adherence may indicate decreased engagement and “behavioral fatigue, “potentially increasing future relapse risk. This underscores the necessity of adaptive, low-burden reinforcement strategies.

Clinical and population-based evidence supports lifestyle modification as fundamental to type 2 diabetes management, with sustained healthy dietary and physical activity patterns associated with improved glycemia and reduced long-term risks (28). Nevertheless, maintaining behavior changes remains challenging in routine care, particularly over extended periods. In our setting, follow-up primarily depended on face-to-face visits, and several behavioral indicators were self-reported, possibly limiting continuity and feedback intensity. Emerging mobile health interventions report improvements in glycemic outcomes, adherence, and patient engagement (29–33). However, sustaining these benefits long-term remains challenging. Integrating scalable digital tools (e.g., tailored messaging, remote monitoring, and feedback loops) into platform-based care may address these gaps and support sustained self-management. As a practical initiative, our institution is exploring a lightweight digital follow-up tool to enhance follow-up efficiency and continuity. Prospective evaluations are required to assess its effectiveness.

### 4.4 Limitations

Several limitations should be acknowledged when interpreting the findings of this study. First, this retrospective, single-center observational design lacked a concurrent control group, limiting the ability to draw causal conclusions about the platform’s effectiveness and increasing the risk of residual confounding and regression to the mean.

Second, outcome ascertainment was incomplete over time. Although most participants had at least one follow-up HbA1c measurement, only a subgroup completed HbA1c assessments at all key visits over the entire 36-month period. To address potential attrition bias, sensitivity analyses were conducted in this subgroup, and baseline characteristics were compared between participants with and without complete follow-up data. Both approaches suggested similar overall trends. While selection bias cannot be entirely excluded, these consistency checks support the reliability of the primary findings.

Third, several secondary measures, including fasting and postprandial glucose and lifestyle behaviors, were predominantly based on patient self-report and collected more frequently (monthly) than HbA1c (approximately every three months in the hospital). Differences in measurement frequency and context could introduce reporting bias and variability, possibly contributing to discrepancies observed between HbA1c and single-point glucose measurements.

Fourth, although the follow-up duration of three years is longer than many previous studies evaluating similar platforms, it might still be insufficient to fully assess the durability of behavioral changes and long-term impacts on complications and clinical outcomes.

Finally, this study was conducted in a tertiary TCM hospital in a single region of China. The platform’s structure and patient characteristics may differ from other healthcare settings, limiting the generalizability of findings. Future multi-center, prospective studies employing standardized exposure measurements and more comprehensive outcome capture are necessary to confirm these results and evaluate hierarchical medical models that integrate tertiary hospital expertise with community-based follow-up.

## 5 Conclusions

In this real-world observational study conducted in a tertiary Traditional Chinese Medicine hospital, sustained glycemic control improvements were observed over 36 months among patients managed via a hospital-based CDM platform. Reductions in HbA1c levels and increased attainment of HbA1c targets were evident compared with baseline. The largest absolute HbA1c reductions occurred in patients with poor baseline control, and clinically significant improvements (≥1.0% HbA1c reduction and sustained target attainment across visits) were common among those with paired measurements. However, improvements in lifestyle behaviors and other metabolic indicators were limited, and adherence to some self-management behaviors declined after the first year.

These findings highlight both the potential and challenges associated with hospital-led CDM within broader health service systems. Future efforts should prioritize sustained behavioral support, more flexible and scalable follow-up facilitated by digital health tools (including mobile health technologies), and enhanced integration with community-based services to improve continuity in long-term diabetes care. Prospective, multi-center studies with standardized exposure measurements and complete outcome assessments are warranted to confirm effectiveness and to identify which platform components most benefit specific patient subgroups.

## Data Availability

The raw data supporting the conclusions of this article will be made available by the authors, without undue reservation.

## Supplementary material

Supplementary Table 1 Number of participants with HbA1c measurements at each key follow-up timepoint.

Supplementary Table 2 Selected baseline characteristics comparing participants with any follow-up HbA1c versus no follow-up HbA1c.

Supplementary Table 3 Glycemic control indicators at baseline and at 12-, 24-, and 36-month follow-ups (n = 112 patients).

Supplementary Table 4 HbA1c control rates at baseline and at 12-, 24-, and 36-month follow-ups (n = 112 patients).

Supplementary Table 5 Lifestyle and health behaviors at baseline and at 12-, 24-, and 36-month follow-ups (n = 112 patients).
