# Supplementary material for: Long-term glycemic outcomes of a diabetes management platform in a traditional Chinese medicine hospital: a real-world retrospective observational study
Source: Front Endocrinol (Lausanne). 2026 Apr 23;17:1799398. doi: 10.3389/fendo.2026.1799398 (PMC13149176; doi:10.3389/fendo.2026.1799398)
Supplement: Supplementary Table 1 — Number of participants with HbA1c measurements at each key follow-up timepoint. [file Table1.docx]

**Supplementary Table S1. Number of participants with HbA1c measurements at each key follow-up time point**

| **Follow-up month** | **N with HbA1c measured** |
| --- | --- |
| 0 | 531 |
| 3 | 375 |
| 6 | 334 |
| 9 | 276 |
| 12 | 328 |
| 24 | 313 |
| 36 | 293 |

**Supplementary Table S2. Selected baseline characteristics comparing participants with any follow-up HbA1c versus no follow-up HbA1c**

| **Variable** | | **Any follow-up HbA1c** | **No follow-up HbA1c** | **P value** |
| --- | --- | --- | --- | --- |
| Sex | Female:274(51.6%);  Male: 257 (48.4%) | | Male: 4 (66.7%);  Female: 2 (33.3%) | 0.439 |
| Age (years) | | 59.41 ± 13.94 | 55.17 ± 10.36 | 0.364 |
| Diabetes duration (years) | | 9.12 ± 6.13 | 4.50 ± 5.61 | 0.100 |
| Baseline HbA1c (%) | | 9.12 ± 2.74 | 8.65 ± 2.38 | 0.648 |
| Baseline BMI (kg/m²) | | 24.16 ± 4.46 | 28.40 ± 3.83 | 0.361 |
| Hypertension | | Missing: 2 (0.4%); No: 265 (49.9%); Yes: 264 (49.7%) | No: 4 (66.7%);  Yes: 2 (33.3%) | 0.713 |
| Comorbidity burden | | ≥3 comorbidities: 290 (54.6%);  <3 comorbidities: 160 (30.1%);  None: 81 (15.3%) | ≥3 comorbidities: 4 (66.7%); None: 2 (33.3%) | 0.200 |
| Prior antidiabetic treatment | | Missing:12 (2.3%);  Yes: 426 (80.2%); No: 93 (17.5%) | Yes: 5 (83.3%);  No: 1 (16.7%) | 0.930 |
| Payment method | | Medical insurance: 406 (76.5%);  Free medical service: 66 (12.4%);  Self-financed medical: 51 (9.6%); Other: 8 (1.5%) | Medical insurance: 5 (83.3%); Self-financed medical: 1 (16.7%) | 0.758 |

Notes: Comorbidity burden is categorized as None, <3 comorbidities, or ≥3 comorbidities. Payment method is categorized as Medical insurance, Free medical service, Self-financed medical, or Other. Continuous variables are presented as mean ± SD; categorical variables as n (%). P-values are from Welch’s t-test (continuous) or chi-square/Fisher’s exact test (categorical), as applicable.

**Supplementary Table S3 Glycemic control indicators at baseline, 12-month, 24-month and 36-month follow-up (112 patients)**

| **Indicator** | **Time point** | **Mean ± SD** | **Adjusted mean (95% CI)** | **Change from baseline (95% CI)** | ***P* value** |
| --- | --- | --- | --- | --- | --- |
| HbA1c (%) | Baseline | 9.95 **±** 2.95 | 9.95 (9.40, 10.5) | Ref. |  |
|  | Month 12 | 7.28 **±** 1.45 | 7.32 (7.02, 7.61) | -2.63 (-3.22, -2.05) | <0.001 |
|  | Month 24 | 7.45 **±** 1.30 | 7.45 (7.15, 7.74) | -2.50 (-3.10, -1.90) | <0.001 |
|  | Month 36 | 7.24 **±** 1.54 | 7.23 (6.90, 7.56) | -2.72 (-3.34, -2.09) | <0.001 |
| FBG (mmol/L) | Baseline | 6.89 **±** 1.54 | 6.85 (6.46, 7.24) | Ref. |  |
|  | Month 12 | 7.07 **±** 1.41 | 7.17 (6.86, 7.48) | 0.32 (-0.13, 0.77) | 0.158 |
|  | Month 24 | 7.50 ± 1.99 | 7.35 (6.90, 7.79) | 0.49 (-0.15, 1.14) | 0.130 |
|  | Month 36 | 7.36 ± 2.21 | 7.35 (6.88, 7.83) | 0.49 (-0.15, 1.14) | 0.094 |
| PBG (mmol/L) | Baseline | 9.04 ± 2.48 | 9.10 (8.42, 9.78) | Ref. |  |
|  | Month 12 | 9.01 ± 2.14 | 8.99 (8.50, 9.48) | -0.11 (-0.91, 0.70) | 0.790 |
|  | Month 24 | 8.20 ± 3.06 | 8.19 (7.42, 8.96) | -0.91 (-1.91, 0.10) | 0.076 |
|  | Month 36 | 8.78 ± 2.74 | 8.79 (8.19, 9.39) | -0.31 (-1.11, 0.49) | 0.446 |
| BMI (kg/m^2^) | Baseline | 23.9 ± 3.58 | 23.9 (23.2, 24.6) | Ref. |  |
|  | Month 12 | 24.3 ± 4.31 | 24.6 (23.5, 25.7) | 0.69 (-0.27, 1.65) | 0.156 |
|  | Month 24 | 23.8 ± 4.53 | 23.7 (22.5, 25.0) | -0.19 (-1.55, 1.18) | 0.786 |
|  | Month 36 | 23.3 ± 6.15 | 23.2 (21.9, 24.6) | -0.69 (-2.14, 0.76) | 0.350 |

**Supplementary Table S4 HbA1c control rate at baseline, 12-month, 24-month and 36-month follow-up (112 patients)**

| **Time point** | **Control rate (95% CI)** | **Change from**  **baseline (95%CI)** | **OR (95% CI)** | **P value** |
| --- | --- | --- | --- | --- |
| Baseline | 0.22 (0.16, 0.31) | Ref. | Ref. |  |
| Month 12 | 0.50 (0.41, 0.60) | 0.28 (0.15, 0.41) | 3.541 (2.216, 5.659) | <0.001 |
| Month 24 | 0.44 (0.33, 0.55) | 0.21 (0.04, 0.39) | 2.708 (1.495, 4.905) | 0.001 |
| Month 36 | 0.50 (0.40, 0.60) | 0.28 (0.12, 0.43) | 3.463 (2.020, 5.936) | <0.001 |

**Supplementary Table S5 Lifestyle and health behaviors** **at baseline, 12-month, 24-month and 36-month follow-up (112 patients)**

| **Indicators** | **Baseline** | **Month 12** | **Month 24** | **Month 36** | **Wald χ²** | **P value** |
| --- | --- | --- | --- | --- | --- | --- |
| Diet (n, %) |  |  |  |  | 14.284 | 0.003^a^ |
| Have control | 60 (80) | 84 (79) | 35 (69) | 51 (61) |  |  |
| No control | 3 (4.0) | 4 (4.0) | 1 (2.0) | 10 (12) |  |  |
| Sometimes eat a lot | 12 (16) | 18 (17) | 15 (30) | 21 (25) |  |  |
| Exercise (n, %) |  |  |  |  | 4.505 | 0.212^b^ |
| Everyday | 56 (50) | 50 (45) | 22 (43) | 30 (36) |  |  |
| >5 times /week | 24 (21) | 27 (25) | 13 (26) | 21 (25) |  |  |
| <4 times /week | 26 (23) | 27 (25) | 11 (22) | 24 (29) |  |  |
| Never | 6 (6.0) | 5 (5.0) | 5 (9.8) | 8 (9.6) |  |  |
| Self-test blood glucose (n, %) |  |  |  |  | 32.023 | <0.001^c^ |
| >1 times /week | 97 (86) | 76 (72) | 28 (55) | 49 (59) |  |  |
| >1 times /month | 13 (12) | 29 (28) | 22 (43) | 30 (36) |  |  |
| <1 times /month | 2 (2.0) | 0 (0) | 1 (2.0) | 4 (4.8) |  |  |
| Mental state (n, %) |  |  |  |  | 2.106 | 0.551^d^ |
| Good | 78 (69) | 73 (67) | 30 (59) | 56 (68) |  |  |
| Slightly anxious | 18 ( 16) | 19 (18) | 13 (26) | 18 (22) |  |  |
| Anxious | 4 (4.0) | 3 (3.0) | 3 (5.9) | 4 (4.8) |  |  |
| Insomnia | 12 (11) | 13 (12) | 5 (9.8) | 5 (6.0) |  |  |
| Medication (n, %) |  |  |  |  | 10.403 | 0.015^e^ |
| Regular | 98 (87) | 82 (77) | 35 (69) | 58 (71) |  |  |
| Sometimes missed | 13 (12) | 25 (23) | 16 (31) | 21 (26) |  |  |
| Often missed | 1 (1.0) | 0 (0) | 0 (0) | 1 (1.2) |  |  |

^a^ Diet: have control vs. others. ^b^ Exercise: everyday vs. others. ^c^ Self-test blood glucose frequency: >1 times /week vs. others. ^d^ Mental state: good vs. others. ^e^ Medication: regular vs. others.
